# Hyperbaric oxygen therapy effectively alleviates D-galactose-induced-age-related cardiac dysfunction via attenuating mitochondrial dysfunction in pre-diabetic rats

**DOI:** 10.18632/aging.202970

**Published:** 2021-04-16

**Authors:** Cherry Bo-Htay, Thazin Shwe, Thidarat Jaiwongkam, Sasiwan Kerdphoo, Wasana Pratchayasakul, Thienchai Pattarasakulchai, Krekwit Shinlapawittayatorn, Siriporn C. Chattipakorn, Nipon Chattipakorn

**Affiliations:** 1 Cardiac Electrophysiology Research and Training Center, Faculty of Medicine, Chiang Mai University, Chiang Mai 50200, Thailand; 2 Cardiac Electrophysiology Unit, Department of Physiology, Faculty of Medicine, Chiang Mai University, Chiang Mai 50200, Thailand; 3 Center of Excellence in Cardiac Electrophysiology Research, Chiang Mai University, Chiang Mai 50200, Thailand; 4 Hyperbaric Oxygen Therapy Center, Faculty of Medicine, Chiang Mai University, Chiang Mai 50200, Thailand

**Keywords:** heart, D-galactose, aging, mirochondria, hyperbaric oxygen therapy

## Abstract

Currently, the prevalence of obesity in aging populations is fast growing worldwide. Aging induced by D-galactose (D-gal) is proven to cause the worsening of cardiac dysfunction in pre-diabetic rats via deteriorating cardiac mitochondrial function. Hyperbaric oxygen therapy (HBOT) has been shown to attenuate D-gal-induced cognitive deterioration through decreased inflammation and apoptosis. We tested the hypothesis that HBOT alleviates D-gal induced cardiac dysfunction via improving mitochondrial function in pre-diabetic rats. Wistar rats (n=56) were fed normal diet or high-fat diet for 12 weeks. For subsequent 8 weeks, they were subcutaneously injected either vehicle (0.9% normal saline) or D-gal (150mg/kg/day). Rats were randomly subdivided into 7 groups at week 21: sham-treated (normal diet fed rats with vehicle (NDV), high-fat diet fed rats with vehicle (HFV), normal diet fed rats with D-gal (NDDg), high-fat diet fed rats with D-gal (HFDg)) and HBOT-treated (HFV, NDDg, HFDg). Sham rats received ambient pressure of oxygen while HBOT-treated ones received 100% oxygen given once daily for 60 minutes at 2 atmosphere absolute. HBOT reduced metabolic impairments, mitochondrial dysfunction and increased autophagy, resulting in an improvement of cardiac function in aged pre-diabetic rats.

## INTRODUCTION

The prevalence of obesity in elderly populations is rising [1, 2] and the prime cause of death among aging population is cardiovascular diseases [3]. Aging is influenced by many factors such as lifestyle, environmental conditions, and genetic predisposition, and the characteristics of aging include telomere attrition, reactive oxygen species (ROS) accumulation, genomic instability and mitochondrial dysfunction [4]. Accumulating evidence has shown that D-gal induced-aging can increase senescence markers, oxidative stress and apoptosis, leading to cardiac dysfunction [5–9]. In addition to aging, chronic high-fat diet consumption is known to induce obesity, insulin resistance, mitochondrial dysfunction and cardiac autonomic imbalance, resulting in cardiac dysfunction [10, 11]. We previously demonstrated that rats receiving high-fat diet for 20 weeks showed significantly reduced cardiac mitochondrial fusion marker mitofusin 2 (MFN2) compared to rats receiving normal diet indicating the mitochondrial dynamics imbalance [12]. Additionally, our recent study indicated that rats receiving high-fat diet for 14 weeks showed impaired mitochondrial dynamics as demonstrated by increased mitochondrial fission and decreased mitochondrial fusion [13]. Moreover, we recently demonstrated that D-gal induced aging exacerbated cardiac dysfunction in pre-diabetic rats via the time-dependent deterioration of mitochondrial function, imbalanced mitochondrial dynamics processes, autophagy, and augmented apoptosis [14]. Therefore, the therapeutic strategies which can alleviate the aggravation of cardiac dysfunction by aging in pre-diabetic condition are still needed.

Numerous therapeutic interventions including various antioxidants and melatonin have been shown to yield advantageous results on the cardiac function in D-gal induced cardiac dysfunction [9, 15, 16]. Hyperbaric oxygen therapy (HBOT) is currently utilized clinically as a standard treatment in patients with carbon monoxide poisoning [17, 18]. Furthermore, previous *in vivo* studies have reported that HBOT could attenuate cognitive impairments via decreasing oxidative stress, inflammation and apoptosis in D-gal induced brains of aging mice [19, 20]. In addition, in obese diabetic rats, HBOT could promote glucose and lipid metabolism in the skeletal muscle, suggesting that HBOT could prevent the increased glucose and adipocyte hypertrophy [21, 22]. In the current study, we sought to test the hypothesis that HBOT effectively alleviates D-gal induced cardiac dysfunction via decreasing metabolic impairments, dysfunctional mitochondria, oxidative stress, inflammation, apoptosis and increasing autophagy in pre-diabetic rats.

## RESULTS

### HBOT ameliorated both D-gal and high-fat diet induced metabolic impairments

Body weight, caloric intake, visceral fat, plasma glucose, insulin, HOMA index, TC, HDL and LDL were assessed not only in sham but also in HBOT-treated rats. All high-fat diet fed rats treated with HBOT and sham showed significantly increased body weight, caloric intake, visceral fat, TC, LDL levels and decreased HDL level, compared to normal diet fed rats: sham-treated normal diet vehicle (NDV), sham-treated normal diet fed rats with D-gal (NDDg) and HBOT-treated NDDg rats (Table 1). However, there was no significant difference in body weight and caloric intake among all high-fat diet fed rats. Additionally, the body weight was not different in the normal diet fed rats between HBOT and sham groups. Furthermore, plasma glucose, TG and food intake were found to be not different among the groups.

**Table 1:** The effect of HBOT on metabolic parameters in pre-diabetic rats after induction of aging by D-gal.

**Parameters**	**Sham**					**HBOT**		
	**NDV**	**HFV**	**NDDg**	**HFDg**		**HFV**	**NDDg**	**HFDg**
Body weight (g)	475 ± 9.3	608.5 ± 11.4^*‡^	477 ± 10.2	621.4 ± 16.5^*‡^		601.8 ± 8.4^*‡^	479 ± 9.6	612.2 ± 7^*‡^
Food intake (g/day)	20.8 ± 0.6	22.4 ± 0.7	21.1 ± 0.5	22.2 ± 0.6		20.4 ± 0.7	20.1 ± 0.5	22.3 ± 0.5
Caloric intake (kcal/day)	82.6 ± 2.4	122.3 ± 3.5 ^*‡^	85.4 ± 2.1	123.6 ± 2.6^*‡^		111.1 ± 3.1^*‡^	81.8 ± 1.6	119.1 ± 2.6^*‡^
Visceral fat (g)	24.6 ± 1.7	58.9 ± 2.7^*‡^	26.6 ± 2.3	59.6 ± 4.1^*‡^		58.5 ± 4.2^*‡^	23.4 ± 1.8	58 ± 7^*‡^
Glucose (mg/dl)	141.3 ± 4.1	135.3 ± 5.4	149.2 ± 5	151.9 ± 7		141 ± 6	135.2 ± 5.1	141.4 ± 3.7
Insulin (ng/ml)	5.7 ± 0.5	9 ± 2.2^*^	10.8 ± 1.3^*^	12.4 ± 1.1^*^		5.6 ± 1.3**^†‡#^**	5.1 ± 0.8**^†‡#^**	5.7 ± 1.7**^†‡#^**
HOMA index	49.4 ± 5.2	83.8 ± 10.2^*^	83.5 ± 8.1^*^	89 ± 6.6^*^		46 ± 10.3^†‡#^	46.4 ± 8.8**^†‡#^**	49.7 ± 13.7**^†‡#^**
Cholesterol (mg/dl)	72.8 ± 5.5	102.8 ± 6.7^*‡^	74.5 ± 4.3	109.6 ± 5.5^*‡^		105.3 ± 11^*‡^	70.7 ± 11.3	103.5 ± 13^*‡^
Triglyceride (mg/dl)	81.3 ± 10.3	103.6 ± 9.3	94.8 ± 3.5	103.3 ± 2.3		95.2 ± 9.8	80.4 ± 10.8	95.1 ± 16.6
HDL (mg/dl)	31.2 ± 1.5	20.4 ± 1.3^*‡^	29 ± 2	19.4 ± 2.6^*‡^		23.4 ± 1.5^*‡^	28.9 ± 2.1	23.6 ± 2.7^*‡^
LDL (mg/dl)	31.1 ± 2.6	50.9 ± 4.5^*‡^	31.8 ± 2.3	55.9 ± 5.9^*‡^		54.1 ± 9.6^*‡^	30.3 ± 6	54.3 ± 7.6^*‡^

Data represent means ± SEM. (n=5/group). *P < 0.05 vs NDV sham, †P < 0.05 vs HFV sham, ‡P < 0.05 vs NDDg sham, #P < 0.05 vs HFDg sham. NDV, normal diet fed rats with vehicle; NDDg, normal diet fed rats with D-gal; HFV, high-fat diet fed rats with vehicle; HFDg, high-fat diet fed rats with D-gal; HOMA, homeostasis model assessment; HDL, high-density lipoprotein; LDL, low-density lipoprotein; HBOT, hyperbaric oxygen therapy.

HBOT-treated HFV, NDDg and HFDg rats showed significant reduction in both plasma insulin and HOMA index when compared to sham-treated HFV, NDDg and HFDg rats, and even restored to the same levels as NDV rats indicating that HBOT improves peripheral insulin sensitivity (Table 1). However, HBOT-treated HFV rats and HFDg rats showed no significant difference in TC, HDL and LDL level, compared to sham-treated HFV and HFDg rats suggesting that HBOT had no effect on lipid metabolism in this model system.

### HBOT efficiently attenuated both D-gal and obesity induced increased cardiac senescence marker expression

**Figure 1:**
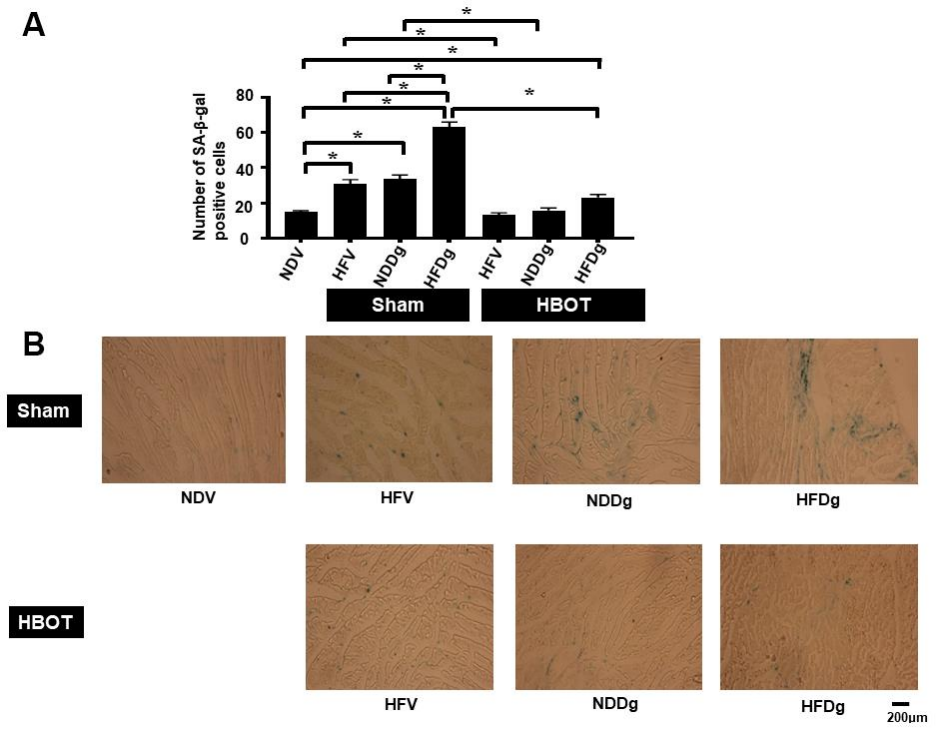
**Effect of HBOT on senescence marker expression in left ventricular cardiomyocytes of pre-diabetic rats after induction of aging by D-gal.** (**A**) Result of SA-β-gal staining. (**B**) Representative figures of SA-β-gal staining. NDV, normal diet fed rats with vehicle; NDDg, normal diet fed rats with D-gal; HFV, high-fat diet fed rats with vehicle; HFDg, high-fat diet fed rats with D-gal; SA-β-gal, senescence associated β galactosidase; HBOT, hyperbaric oxygen therapy. (n = 3/group). ^*^P < 0.05.

SA-β-gal staining was determined as senescence marker expression in cardiomyocytes. The results showed that HBOT-treated HFV rats and NDDg rats had significantly decreased number of SA-β-gal positive cells compared to sham-treated HFV rats, NDDg rats and even restored to the same level as NDV rats (Figure 1A, 1B). Additionally, HBOT-treated HFDg group had significantly reduced senescence marker cells in cardiomyocytes when compared to its respective sham HFDg indicating that HBOT effectively attenuates increased cardiomyocytes senescent marker expression in pre-diabetic rats after the induction of aging by D-gal (Figure 1A, 1B).

### HBOT effectively ameliorated LV dysfunction in pre-diabetic rats after induction of aging by D-gal

**Figure 2:**
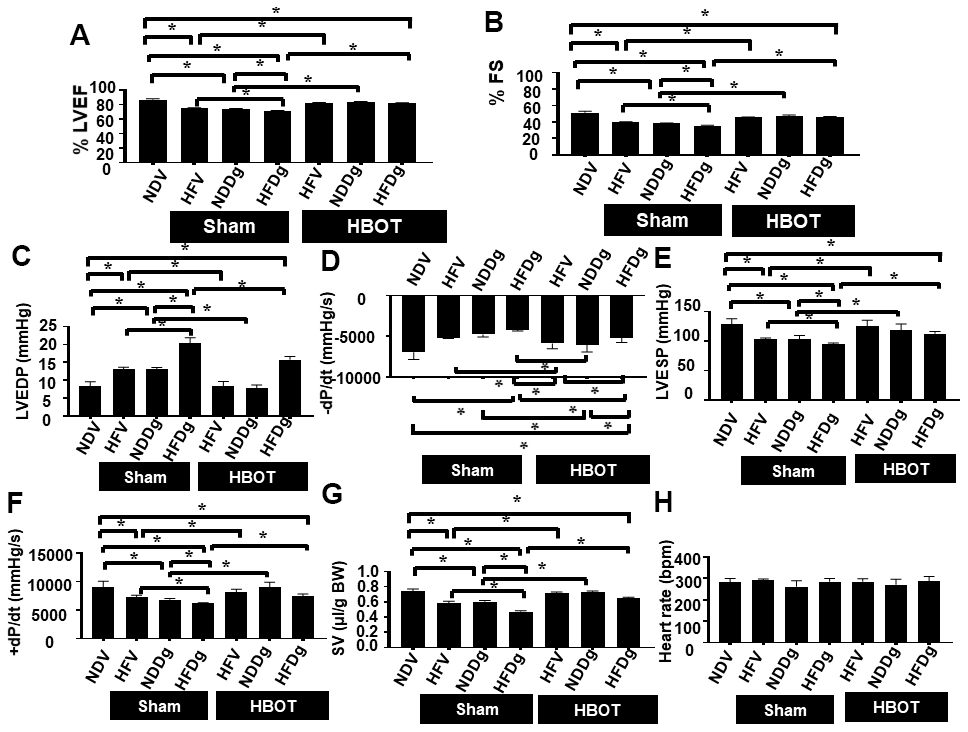
**Effect of HBOT on cardiac function in pre-diabetic rats after induction of aging by D-gal.** (**A**) Ejection fraction. (**B**) Fractional shortening. (**C**–**H**) P-V loop Analysis. NDV, normal diet fed rats with vehicle; NDDg, normal diet fed rats with D-gal; HFV, high-fat diet fed rats with vehicle; HFDg, high-fat diet fed rats with D-gal; EF, ejection fraction; FS, Fractional shortening; HBOT, hyperbaric oxygen therapy. (n = 8/group). ^*^P < 0.05.

Sham-treated HFV, NDDg and HFDg rats showed significantly decreased %EF and %FS compared to sham NDV rats. Additionally, sham-treated HFDg rats had significantly impaired LV function as shown by significantly decreased %EF and %FS, compared with HFV sham and NDDg sham rats (Figure 2A, 2B). In contrast, HBOT-treated HFDg rats revealed significantly increased %EF and %FS, when compared to sham-treated HFDg rats. Interestingly, HFV and NDDg rats treated with HBOT displayed significantly increased %EF and %FS compared to sham-treated HFV, NDDg rats and even restored to the same %EF and %FS levels as NDV rats (Figure 2A, 2B).

**Figure 3:**
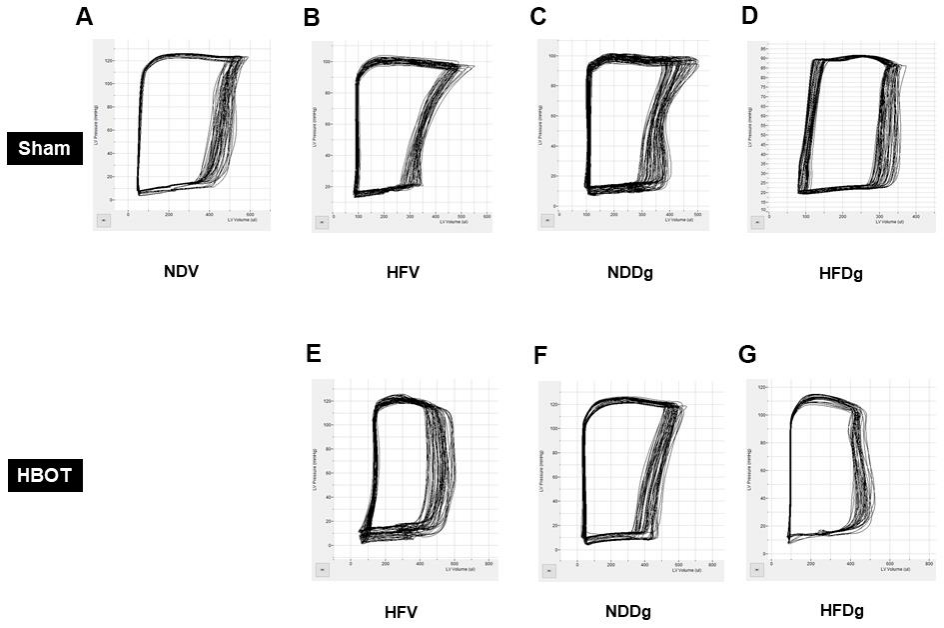
**Effect of HBOT on typical P-V loop tracings in pre-diabetic rats after induction of aging by D-gal.** (**A**) A typical P-V loop tracing of sham-treated NDV rat. (**B**) A typical P-V loop tracing of sham-treated HFV rat. (**C**) A typical P-V loop tracing of sham-treated NDDg rat. (**D**) A typical P-V loop tracing of sham-treated HFDg rat. (**E**) A typical P-V loop tracing of HBOT-treated HFV rat. (**F**) A typical P-V loop tracing of HBOT-treated NDDg rat. (**G**) A typical P-V loop tracing of HBOT-treated HFDg rat. NDV, normal diet fed rats with vehicle; NDDg, normal diet fed rats with D-gal; HFV, high-fat diet fed rats with vehicle; HFDg, high-fat diet fed rats with D-gal.

Pressure-volume (P-V) loop analysis was determined at the end of the study protocol, and sham-treated HFDg rats indicated the worst deterioration of LV function as indicated by significantly increased LVEDP (Figure 2C), dP/dt min (Figure 2D) and significantly reduced LVESP (Figure 2E), dP/dt max (Figure 2F) and SV (Figure 2G), compared to sham-treated rats. In contrast, HBOT treatment effectively alleviated LV dysfunction in HFDg rats as shown by significantly decreased LVEDP, dP/dt min and significantly increased LVESP, dP/dt max and SV when compared to sham-treated HFDg rats (Figure 2C–2G). Similar to echo results, HFV and NDDg rats treated with HBOT had significantly reduced LVEDP, dP/dt min and significantly increased LVESP, dP/dt max and SV, when compared to sham-treated HFV, NDDg rats, and restored the above parameters to the same level as NDV rats (Figure 2C–2G). However, there was no significant difference in HR among all groups (Figure 2H). The representative typical P-V loop tracing of all experimental groups were shown in Figure 3A–3G.

**Figure 4:**
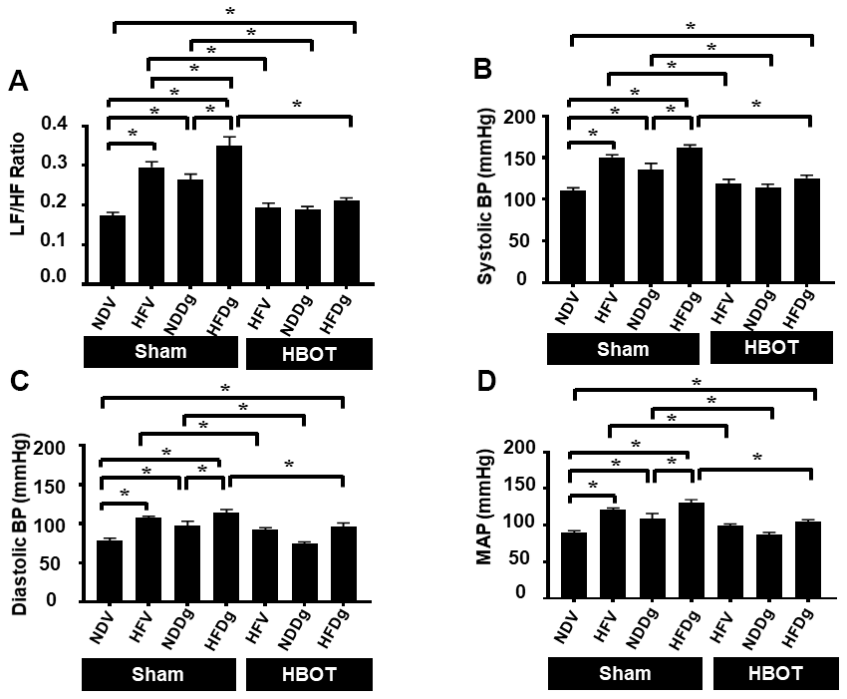
**Effect of HBOT on heart rate variability and blood pressure in pre-diabetic rats after induction of aging by D-gal.** (**A**) Heart rate variability. (**B**) Systolic blood pressure. (**C**) Diastolic blood pressure. (**D**) Mean arterial blood pressure. NDV, normal diet fed rats with vehicle; NDDg, normal diet fed rats with D-gal; HFV, high-fat diet fed rats with vehicle; HFDg, high-fat diet fed rats with D-gal; LF/HF, low frequency/high frequency ratio; SBP, systolic blood pressure; DBP, diastolic blood pressure; MAP, mean arterial pressure; HBOT, hyperbaric oxygen therapy. (n = 8/group). ^*^P < 0.05.

Regarding HRV measurement, LF/HF ratio was evaluated as a cardiac sympathovagal balance indicator. Sham-treated HFV, NDDg and HFDg rats had significantly increased LF/HF ratio, compared with the NDV sham group. In addition, HFDg sham rats had higher LF/HF ratio compared with the HFV and NDDg sham. The results also showed that HFV and NDDg rats treated with HBOT had restored LF/HF ratio as the same level as NDV rats. Additionally, HBOT-treated HFDg rats had significantly reduced LF/HF ratio, when compared with its respective sham rats (Figure 4A). For blood pressure (BP) assessment; HBOT-treated HFV and NDDg rats showed significant decrease in systolic, diastolic and mean arterial pressure when compared to sham-treated HFV and NDDg rats. Likewise, HFDg rats treated with HBOT had significantly reduced SBP, DBP and MAP compared to sham-treated HFDg rats (Figure 4B–4D).

### HBOT restored cardiac apoptosis and inflammation to normal levels in D-gal induced aging or obese rats, and attenuated such impairments in aging pre-diabetic rats

**Figure 5:**
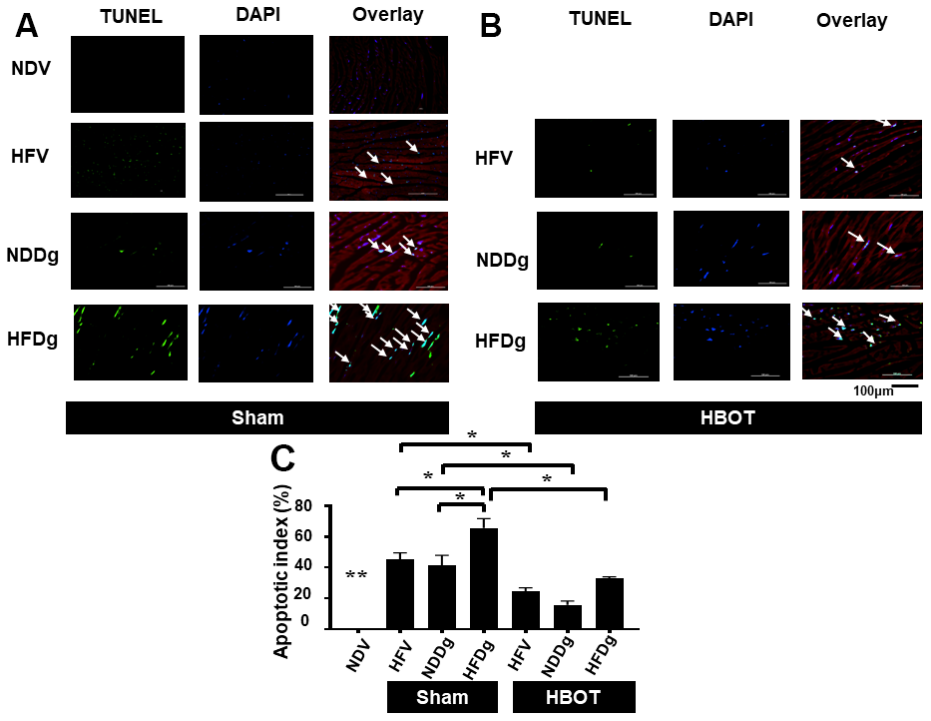
**Effect of HBOT on apoptosis using TUNEL staining in cardiomyocytes of pre-diabetic rats after induction of aging by D-gal.** (**A**) Representative figure of TUNEL positive cells in sham-treated rats. (**B**) Representative figure of TUNEL positive cells in HBOT-treated rats. (**C**) Percentage of apoptotic index. NDV, normal diet fed rats with vehicle; NDDg, normal diet fed rats with D-gal; HFV, high-fat diet fed rats with vehicle; HFDg, high-fat diet fed rats with D-gal; TUNEL, Terminal deoxynucleotidyl transferase dUTP nick end labeling; HBOT, hyperbaric oxygen therapy. (n = 3/group). ^*^P < 0.05, ^**^P < 0.01 compared to other groups.

**Figure 6:**
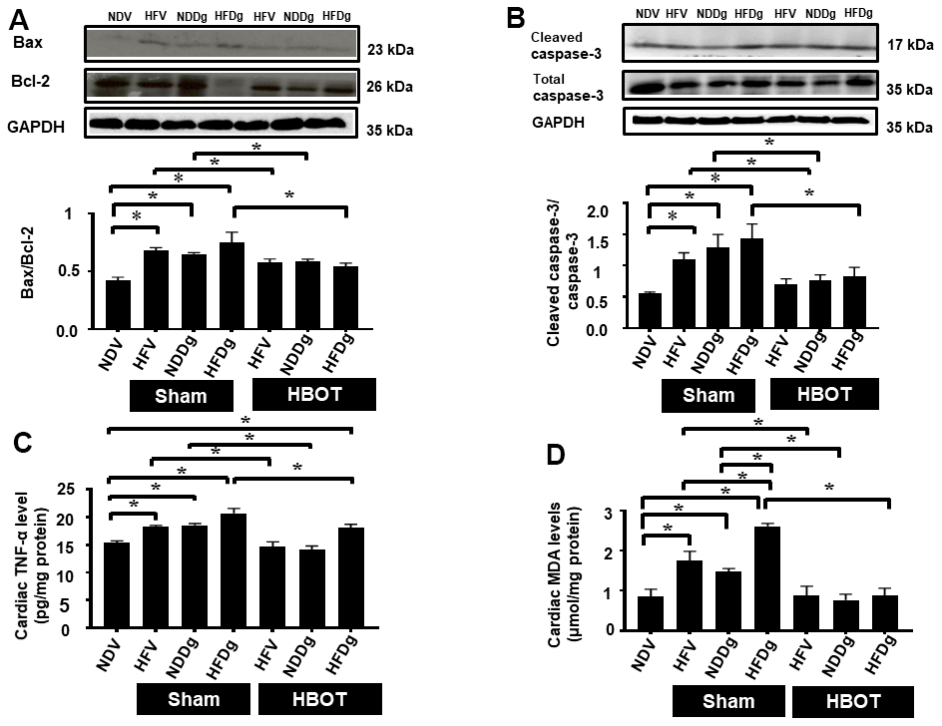
**Effect of HBOT on cardiomyocytes apoptosis, inflammation and oxidative stress in pre-diabetic rats after induction of aging by D-gal.** (**A**) Bax/Bcl-2 ratio. (**B**) Cleaved caspase-3/caspase-3 ratio. (**C**) Inflammation. (**D**) Oxidative stress. NDV, normal diet fed rats with vehicle; NDDg, normal diet fed rats with D-gal; HFV, high-fat diet fed rats with vehicle; HFDg, high-fat diet fed rats with D-gal; Bax, B-cell lymphoma 2 associated X protein; Bcl-2, B-cell lymphoma 2; TNF-α, tumor necrosis factor alpha; MDA, malondialdehyde; HBOT, hyperbaric oxygen therapy. (n = 5/group). ^*^P < 0.05.

For the determination of cardiac cell apoptosis, TUNEL assay, Bax/Bcl-2 ratio and cleaved-caspase 3/caspase 3 ratio and were performed. Our results demonstrated that, TUNEL positive apoptotic cells were significantly decreased in HBOT-treated HFV and NDDg rats compared to sham-treated HFV and NDDg rats. HBOT-treated HFDg rats had significantly reduced TUNEL positive cells when compared to sham-treated HFDg rats (Figure 5A–5C). Furthermore, Bax/Bcl-2 ratio and cleaved caspase 3/caspase 3 ratio were significantly reduced in all HBOT-treated rats compared to their respective sham-treated rats (Figure 6A, 6B). Cardiac TNF-α level was used as an inflammatory marker, and only HBOT-treated HFV and NDDg rats had restored TNF-α level to the same level as NDV rats. HBOT also attenuated the increased cardiac inflammation in HFDg rats, compared to sham-treated HFDg rats (Figure 6C).

### HBOT reduced D-galactose-induced aggravation of increased cardiac oxidative stress in pre-diabetic rats

For oxidative stress status, cardiac tissue MDA was determined and the results indicated that sham-treated HFV, NDDg and HFDg rats showed significantly increased cardiac MDA level than those sham-treated NDV rats, among which sham-treated HFDg rats had higher MDA level than sham-treated HFV and NDDg rats. Interestingly, all HBOT-treated rats had significantly decreased cardiac MDA level compared to their respective sham groups and even restored to the same MDA level as NDV rats (Figure 6D).

### HBOT ameliorates D-gal induced aggravation of mitochondrial dysfunction in pre-diabetic rats

**Figure 7:**
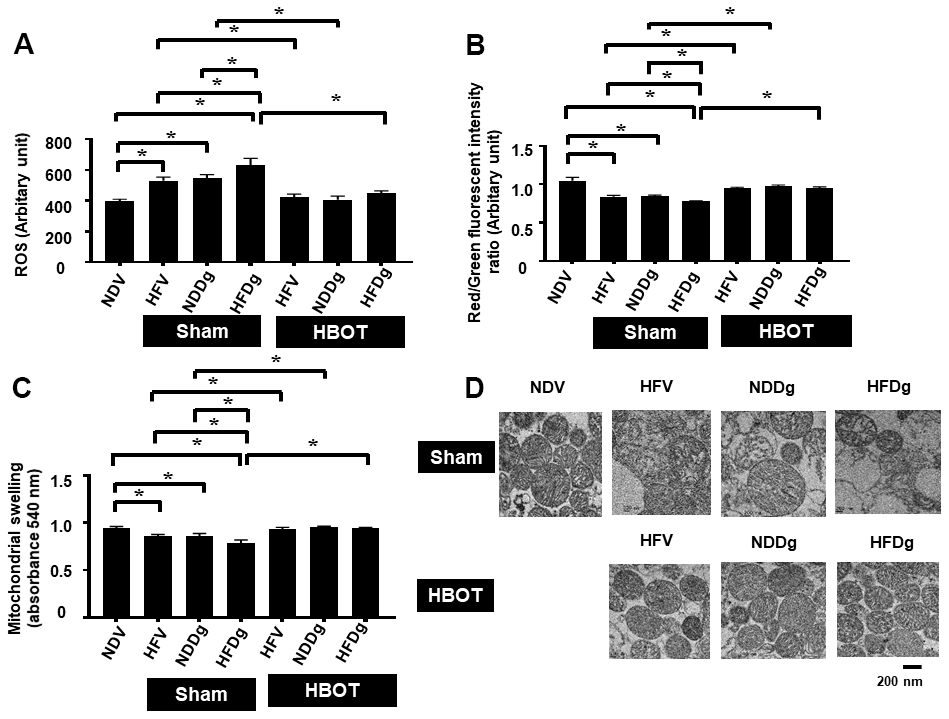
**Effect of HBOT on mitochondrial functions in cardiomyocytes of pre-diabetic rats after induction of aging by D-gal.** (**A**) Cardiac mitochondrial ROS production. (**B**) Cardiac mitochondrial membrane potential. (**C**) Cardiac mitochondrial swelling. (**D**) TEM representative images of cardiac mitochondria. NDV, normal diet fed rats with vehicle; NDDg, normal diet fed rats with D-gal; HFV, high-fat diet fed rats with vehicle; HFDg, high-fat diet fed rats with D-gal; ROS, reactive oxygen species; TEM, transmission electron microscopy; HBOT, hyperbaric oxygen therapy. (n = 8/group). ^*^P < 0.05.

Mitochondrial ROS production, depolarization, and swelling were evaluated as mitochondrial function assessment. Among sham-treated rats, HFDg rats showed the worst deterioration of mitochondrial function as revealed by increased mitochondrial ROS level, depolarization, and swelling. After HBOT, all HFV, NDDg and HFDg rats showed significantly reduced ROS production, depolarization, and swelling of mitochondria compared to their respective sham rats (Figure 7A–7C). Additionally, the morphology of cardiac mitochondria as represented by transmission electron micrographs (TEM) revealed that swelling of mitochondria in sham-treated HFV, NDDg rats and HFDg rats, as indicated by unfolding of cristae, were abolished after HBOT and even restored to normal morphology as NDV rats (Figure 7D).

### HBOT failed to attenuate the impaired mitochondrial dynamics processes in aging pre-diabetic rats

**Figure 8:**
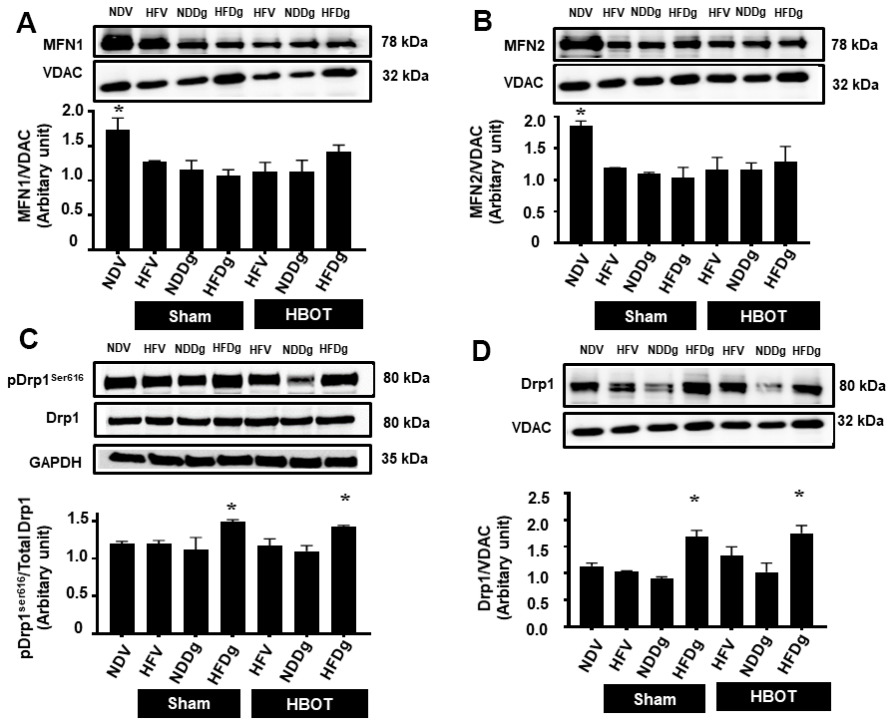
**Effect of HBOT on mitochondrial dynamics parameters in cardiomyocytes of pre-diabetic rats after induction of aging by D-gal.** (**A**) Mitochondrial MFN1 level. (**B**) Mitochondrial MFN2 level. (**C**) Phosphorylated Drp1 at serine 616 in cytosol. (**D**) Mitochondrial Drp1 level. NDV, normal diet fed rats with vehicle; NDDg, normal diet fed rats with D-gal; HFV, high-fat diet fed rats with vehicle; HFDg, high-fat diet fed rats with D-gal; MFN1, mitofusin 1; MFN2, mitofusin 2; Drp1, dynamin-related protein 1; VDAC, voltage-dependent anion channels; HBOT, hyperbaric oxygen therapy. (n = 5/group). ^*^P < 0.05 compared to other groups.

For mitochondrial fusion, MFN1 and MFN2 protein expressions were assessed. MFN1 and MFN 2 expressions were significantly decreased in both sham-treated HFV, NDDg, HFDg rats and HBOT-treated HFV, NDDg and HFDg rats, compared to NDV rats (Figure 8A, 8B). Regarding the process of mitochondrial fission, the phosphorylated form of cytosolic Drp1 expression at serine 616 (Figure 8C) and the mitochondrial fraction (Figure 8D) were evaluated. Our results showed that sham-treated HFDg rats had significantly increased cytosolic phosphorylated Drp1 expression and mitochondrial Drp1 expression compared with the NDV rats, and HBOT could not attenuate this increased Drp1 expression in HFDg rats (Figure 8C, 8D).

### HBOT effectively alleviated the D-gal induced impairment of autophagy in pre-diabetic rats

**Figure 9:**
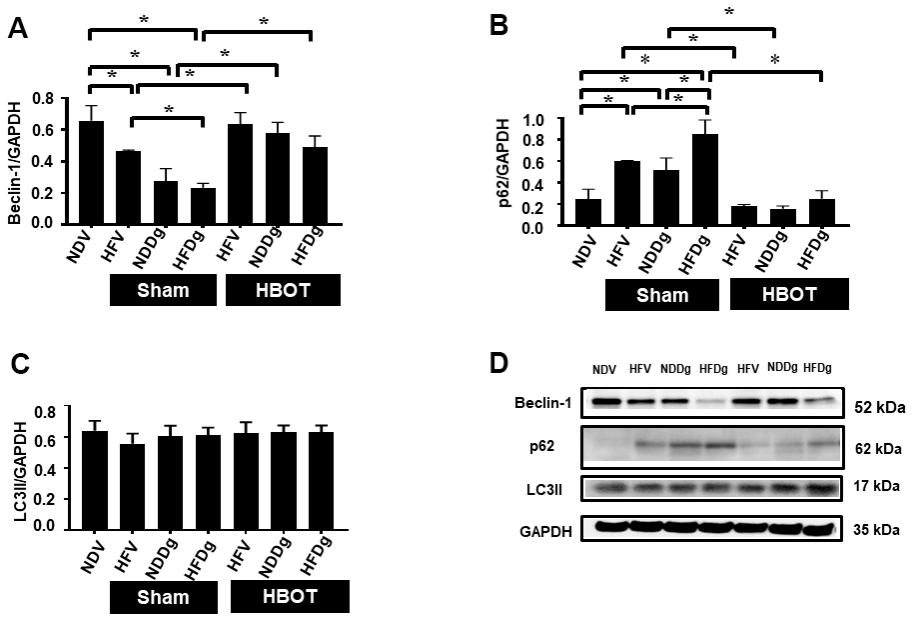
**Effect of HBOT on autophagy in cardiomyocytes of pre-diabetic rats after induction of aging by D-gal.** (**A**) Beclin-1 expression. (**B**) p62 expression. (**C**) LC3II expression. (**D**) Representative images of western blotting bands. NDV, normal diet fed rats with vehicle; NDDg, normal diet fed rats with D-gal; HFV, high-fat diet fed rats with vehicle; HFDg, high-fat diet fed rats with D-gal; p62, Sequestosome-1 (ubiquitin-binding protein); LC3II, microtubule associated light chain 3II; HBOT, hyperbaric oxygen therapy. (n = 5/group). ^*^P < 0.05.

Cardiac autophagic processes were assessed by determination of Beclin-1, p62 and LC3II. The findings indicated that a significant reduction of Beclin-1 and an increased p62 were observed in sham-treated HFV, NDDg and HFDg rats compared to NDV rats. In contrast, all HBOT-treated groups showed significantly increased Beclin-1 and decreased p62 compared to their respective sham rats and even restored the same Beclin-1 and p62 level as NDV rats (Figure 9A, 9B, 9D). However, there was no significant difference in LC3II level among the groups (Figure 9C, 9D).

## DISCUSSION

The major findings from this study demonstrated that D-gal induced aging impaired cardiac function via increased cardiac oxidative stress, inflammation, apoptotic cells, metabolic and autophagic impairments, mitochondrial dysfunction, autonomic and mitochondrial dynamics imbalance. Secondly, these impairments were aggravated in high-fat diet induced pre-diabetic rats after induction of aging by D-gal. Thirdly, HBOT effectively restored the normal cardiac functions in high-fat vehicle rats (HFV) and normal diet fed rats with D-gal (NDDg) as the same level as in NDV rats. Finally, HBOT efficiently ameliorated the aggravation of cardiac dysfunctions in pre-diabetic rats after D-gal-induced aging.

### D-gal induced aging and cardiometabolic status

In the present study, D-gal was used to induce aging process in rats. After injected with D-gal at a dose of 150 mg/kg per day for 8 weeks, the insulin resistance was induced in normal diet fed rats as revealed by increased insulin level and HOMA index compared to NDV rats, and the SA-β-gal positive cells were significantly increased in NDDg rats. Accumulating evidence suggests that administration of D-gal led to cardiac dysfunctions due to increased oxidative stress, apoptotic cells, inflammation and decreased autophagy [6–8, 14, 23, 24]. In the present study, we demonstrated that D-gal administration for 8 weeks significantly increased oxidative stress, increased inflammation and sympathetic overactivity, increased apoptosis and decreased autophagy. In addition, NDDg rats had also developed mitochondrial dysfunctions as demonstrated by increased mitochondrial ROS production, depolarization, swelling, and reduced mitochondrial fusion (MFN1 and MFN2) compared to sham-treated NDV rats. All of the above impairments led to cardiac dysfunction as demonstrated by decreased %EF and %FS in NDDg rats (Table 2). In addition to aging, obesity is also the notable risk factor to induce the cardiac dysfunction. Furthermore, brown adipose tissue (BAT) has metabolic functions such as secretion of BATokines, thermogenesis and serves as metabolic sink for glucose and lipids resulting in decreased obesity and increased insulin sensitivity. BAT mass and activity decreases with aging and associated with metabolic syndrome and age-related diseases [25]. Accumulating evidence has shown that long-term high-fat diet fed pre-diabetic rats developed obesity and insulin resistance, and had mitochondrial dysfunction, cardiac autonomic imbalance, leading to cardiac dysfunction [10, 11]. Additionally, we have previously shown that high-fat diet fed rats for 16 weeks showed an increase in oxidative stress, apoptosis, impaired mitochondrial function when compared to normal diet fed counterparts, resulting in LV dysfunction, and more deterioration of LV dysfunction was observed in high-fat diet receiving rats for 20 weeks [14, 26]. Consistently, our data also revealed that sham-treated HFV rats showed significant increase in oxidative stress, inflammation, apoptotic cells and impaired mitochondrial function resulting in deterioration of cardiac function when compared to sham-treated NDV rats.

**Table 2:** Summarized effect of HBOT on cardiometabolic impairment in pre-diabetic rats after induction of aging by D-gal.

**Cardiometabolic impairments**	**Sham**					**HBOT**		
	**NDV**	**HFV**	**NDDg**	**HFDg**		**HFV**	**NDDg**	**HFDg**
Metabolic disturbance	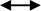	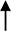	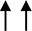	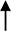		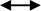	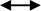	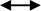
Aging Marker	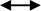	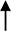	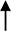	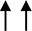		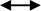	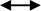	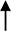
Autonomic imbalance	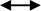	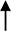	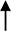	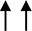		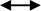	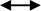	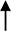
LV dysfunction	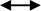	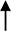	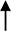	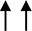		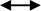	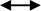	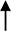
Mitochondrial impairment	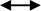	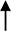	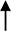	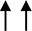		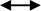	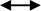	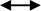
Inflammation	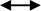	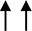	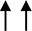	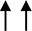		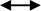	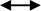	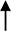
Apoptosis	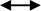	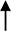	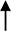	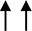		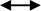	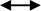	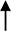
Oxidative stress	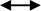	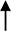	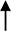	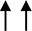		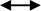	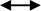	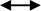
Autophagy	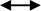	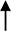	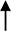	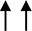		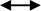	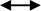	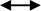

NDV, normal diet fed rats with vehicle; NDDg, normal diet fed rats with D-gal; HFV, high-fat diet fed rats with vehicle; HFDg, high-fat diet fed rats with D-gal**;** HBOT, hyperbaric oxygen therapy.

One up arrow indicates impairment of cardiometabolic parameters, two up arrows indicate severe impairment of cardiometabolic parameter and double headed arrow indicates no significant change in cardiometabolic parameters compared to NDV rats.

### Prediabetes with aging induced by D-gal and cardiometabolic status

Currently, the occurrence of obese-insulin resistance is rising sharply amongst adolescents and adults throughout the world. However, the effects of D-gal induced aging on cardiometabolic impairments in pre-diabetic condition are not well-established. The current study investigated the effect of D-gal induced aging on cardiometabolic parameters in high-fat diet induced pre-diabetic rats. In accordance with our recent report [14], we found that HFDg rats developed insulin resistant condition about the same level as NDDg rats (Table 1) and impaired lipid metabolism as indicated by increased total cholesterol level, LDL level and decreased HDL level compared to NDV rats. Moreover, HFDg rats showed an increase in oxidative stress, dysfunctional mitochondria, inflammation, apoptotic cells and decreased autophagy when compared to NDV rats, and these impairments were much more aggravated than HFV and NDDg rats, leading to the exacerbation of cardiac dysfunction in HFDg rats (Table 2). Therefore, highly effective strategies and novel alternative treatment are required to prevail over the bad consequences of this elderly pre-diabetic condition.

### HBOT intervention in D-gal induced aging and cardiometabolic improvement

The therapeutic efficacy of hyperbaric oxygen therapy (HBOT) is widely observed in carbon monoxide poisoning and O_2_ toxicity is rare in clinical use of HBOT [27]. Furthermore, previous studies have demonstrated that HBOT significantly reverses D-gal induced learning and memory impaired mice by upregulating antioxidant enzymes and reducing oxidative stress, inflammation, apoptosis and aging-related proteins expression [19, 20]. Therefore, we sought to evaluate the effect of HBOT on cardiometabolic parameters in D-gal induced cardiac aging rats. We found that HBOT significantly alleviated metabolic impairments in NDDg rats as indicated by decreased plasma insulin level and HOMA index compared to sham-treated NDDg rats, and even restored to the same level as NDV rats in the present study (Table 1). The roles of HBOT on senescence markers have been reported. It has been shown that HBOT effectively decrease hippocampal senescence marker expressions (p21 and p53) in D-gal induced brain aging mice [20]. In our study, HBOT significantly reduced cardiac senescence marker expression in NDDg and even restored to the same level of NDV rats (Table 2). These findings suggested that HBOT could attenuate the cardiometabolic impairments and reduced the aging process in the heart in D-gal induced aging rats.

The mechanism responsible for these benefits might be attributable to the findings that HBOT could reduce the ROS production, depolarization, and swelling of the cardiac mitochondria in NDDg rats compared to its respective sham-treated rats, and had the similar levels of mitochondrial functions and morphology as found in NDV rats (Table 2). A recent study has shown that HBOT could lead to a production of enzymatic and non-enzymatic antioxidants, resulting in decreased oxidative stress and damage [27]. In addition, through restoration of oxygen tension and cellular energy production, HBOT increased mitochondrial oxidative phosphorylation, ATP production and decreased mitochondrial DNA damage [28]. In rats with focal brain injury, HBOT (100% O_2_, 2.8 atmosphere absolute (ATA) for 45 minutes, twice per day for 3 days) could provide neuroprotective effect through decreasing mitochondrial membrane depolarization and apoptotic markers [29]. These could lead to improved mitochondrial function in HBOT treated NDDg rats in our study. In addition, HBOT effectively decreased apoptosis and increased autophagy in NDDg rats compared to its sham-treated counterpart, and even restored them to the same apoptotic level and autophagy level as found in NDV rats (Table 2). Consistently, a recent study has shown that HBOT (2ATA, 60 minutes, once daily for 5 days) significantly increased autophagic flux as indicated by increased LC3-II and decreased p62 via inhibiting the mechanistic target of the rapamycin (mTOR) pathway in rats with spinal nerve ligation (SNL) compared to sham-SNL rats [30]. Contrary to our findings, HBOT preconditioning (2.5ATA, 1hr, one time per day for 14days) could reduce Beclin-1 and increased mTOR expression resulting in the inhibition of exaggerated autophagy in rats with myocardial ischemia reperfusion injury [31]. The discrepancy between a previous study and ours might be owing to the difference between the models (MIRI vs aging rats) and the pressure used for HBOT (2.5ATA vs 2ATA). Therefore, our study demonstrated that HBOT effectively provided cardiometabolic protection through attenuation of cardiac oxidative stress, inflammation, apoptotic cells, dysfunctional mitochondria, and increased autophagy in NDDg rats, leading to the restoration of cardiac functions in the present study (Table 2). Consistently, a recent clinical study has demonstrated that %LVEF from echocardiographic result was significantly increased in elderly men after receiving HBOT (2ATA for 90 minutes (with 5 minutes air breaks every 20 minutes), once a day, 5days per week for 60days) when compared to %LVEF before receiving HBOT [32].

### HBOT intervention in prediabetes and prediabetes with aging and cardiometabolic improvement

In pre-diabetic rats, our results demonstrated that HBOT decreased oxidative stress, inflammation, apoptotic cells and dysfunctional mitochondria compared to sham-treated HFV rats leading to effectively restored cardiac functions (Table 2). Furthermore, HBOT also ameliorated glucose metabolism impairments as revealed by decreased plasma insulin level and HOMA index in pre-diabetic rats compared to sham-treated HFV rats and even restored to the same level as NDV rats (Table 1). Consistently, a previous study has shown that streptozotocin induced diabetic rats which received HBOT (2.3ATA for 1 h/day) for 10 days showed significantly reduced blood glucose level which was associated with significant decrease in the percentage of β-cell damage compared to diabetic rats without HBOT [33]. In diabetic rats with obesity, HBOT could enhance the metabolism of glucose and lipid in the skeletal muscle, suggesting that HBOT can prohibit an elevated glucose level and adipocyte hypertrophy [22]. Additionally, a previous study demonstrated reduced blood glucose and insulin levels, increased skeletal IL-10 level and decreased adipose tissue TNF-α level in HBOT (1.3ATA) treated obese type 2 diabetic rats when compared with no HBOT rats [21].

In pre-diabetic aging rats, the worsening of cardiac dysfunction was found, compared to either aging rats or pre-diabetic rats as demonstrated in the present study. To attenuate these detrimental effects of combined aging and prediabetes, the HBOT was used to alleviate the adverse effects of such combined conditions. Regarding metabolic impairments, HBOT only effectively attenuated the increased plasma insulin level and HOMA index in HFDg rats compared to sham-treated HFDg rats (Table 1). However, the increased total cholesterol level, LDL level and decreased HDL level found in HFDg rats were not alleviated by HBOT (Table 1). Consistent with a previous study, treatment with HBOT (2.5ATA for 60minutes per day) for 2weeks, 2 cycles in MSG induced obese mice showed no significant difference in total cholesterol, LDL and HDL levels when compared to that of the sham-treated MSG mice [34]. Our findings indicated that HBOT-treated high-fat diet D-gal (HFDg) rats had significantly reduced cardiac oxidative stress, metabolic and autophagic impairments and mitochondrial dysfunction compared to sham-treated HFDg rats, and even restored them to the same levels as NDV rats. However, although HBOT-treated HFDg rats showed significantly increased LV functions compared to its sham-treated rats, it did not reach the same level of cardiac function found in NDV rats. This may be explained by that fact that the severity of mitochondrial dysfunctions and cardiac impairments was worse in sham-treated HFDg rats, compared to sham-treated HFV and NDDg rats. Moreover, mitochondrial dynamics impairments in HFDg rats were not diminished after HBOT. It is well-known that cellular senescence is a notable factor which contributes to inflammation and accelerates the aging process [35, 36]. A growing body of evidence has revealed that senescent cells could yield inflammatory cytokines, for instance TNF-α, IL-6 resulting in mitochondrial dynamics impairments [35, 37, 38]. Cardiac senescent cells were indicated as an increased expression of SA-β-gal positive cells and cardiac inflammation was demonstrated by an increased expression of TNF-α level in the heart in the current study. HBOT could only alleviate the increased SA-β-gal positive cells and cardiac TNF-α in HFDg rats, but could not bring back the senescent and inflammatory level to the same as that of the NDV rats (Table 2). These results might be one of the reasons why HBOT could not mitigate the impairments of mitochondrial dynamics in HFDg rats. Additionally, rats in the current study received HBOT only at 2ATA for 1hr, one time per day for 2weeks. However, earlier experiments of D-gal induced brain aging mice received HBOT at 2.5ATA for 1hr, one time per day for 2weeks [19, 20]. Furthermore, HBOT preconditioning at 2.5ATA, 1hr, one time per day for 2weeks could effectively decrease infarct size compared to sham-treated rats [39]. Hence, the increased severity of impairments found in high-fat D-gal rats and the variation in given HBOT pressure might be the reasons why HBOT could not bring back the cardiac function level to the same level as NDV rats in HFDg rats. Consequently, HBOT could only decrease inflammation and apoptotic cells in HFDg rats compared to sham-treated HFDg rats, but not reach the same level to that of NDV rats (Table 2).

## CONCLUSIONS

Our results indicated that HBOT could restore the normal cardiac function in either aging induced by D-gal rats or high-fat diet induced pre-diabetic rats. Mechanistically, HBOT effectively alleviated the cardiac dysfunction in aging pre-diabetic rats through decreased oxidative stress, inflammation, apoptosis, dysfunctional mitochondria, metabolic and autophagic impairments. Thus, HBOT could be a potential therapeutic intervention in aging pre-diabetic people with impaired cardiac function.

### Limitations

From a clinical translation point of view, the HBOT-treated NDV group was not performed in the present study. In clinical settings particularly, HBOT is prescribed for patients under specific pathological conditions. In addition, a recent study has indicated that glucose profile, plasma MDA level and cardiac functions were not different between HBOT-treated control rat and no HBOT-control rat [40]. Regarding the hallmarks of alterations of cardiac structures, an earlier investigation has shown that cardiac architecture was not altered according to H and E staining. Furthermore, Masson’s Trichrome staining revealed that cardiac fibrosis was not observed in D-gal injected mice when compared to their control counterparts [6]. As a result, we did not determine cardiac collagen deposits and coronary density in the present study. In this study, the arterial blood gas was not measured to verify the change of PaO. However, a previous HBOT related investigation has revealed that arterial blood was collected through right carotid puncture in the model of acute lung injury rats with HBOT treatment [41], and the results revealed that PaO_2_ was significantly increased and PaCO_2_ was decreased in HBOT treated rats compared to non-HBOT rats. Consistently, an earlier study has described that HBOT treated rats showed significantly increased PaO and decreased PaCO_2_ compared to non-HBOT rats in carbon dioxide poisoning rat model [42]. These findings demonstrated the significant changes in improved arterial blood gas due to HBOT.

In addition, although this study did not investigate the dose-dependent effects of HBOT, previous studies have already demonstrated it in different models. In the rat model of burn-induced neuropathic pain, rats treated with two-week HBOT had lower expressions of the pain-related neuropeptides (Substance P and CGRP) in the hind paw skin compared to rats treated with HBOT for one-week rats [43]. Future studies are needed to investigate the dose-dependent effects for an optimal HBOT dose with greatest protective efficacy. ROS is known to affect cardiac pathological remodeling, and could alter cardiomyocyte size and degree of fibrosis in cardiac tissues. However, we did not determine these parameters. Future studies need to determine whether HBOT effectively alter cardiomyocyte size and fibrosis in this model.

## MATERIALS AND METHODS

### Animals

All rats were conducted in accordance with ethical procedures approved by Faculty of Medicine, Chiang Mai University, Thailand (Institutional Animal Care and Use Committee, with the approval number. 43/2562). Fifty-six male Wistar rats weighing 200-220 g were acquired from Bangkok, Thailand (Nomura Siam International Co., Ltd.). The rats were provided with normal rat chow, water ad libitum.

### Experimental design and hyperbaric oxygen therapy (HBOT)

One week after acclimatization period, male Wistar rats were allocated to receive a normal diet (ND) with 19.77% energy, as well as a high-fat diet (HFD) with 52.98% energy, for 12 weeks to induce obese-insulin resistant condition [44]. After that, rats were separated into a vehicle group (0.9% normal saline, subcutaneous injection (SC)) and a D-gal group (150 mg per kg/day, SC) for further 8 weeks and continued their respective diets; ND and HFD up to week 20. Rats were further randomly subdivided into sham-treated and HBOT-treated rats at week 21. For sham-treated rats, they were divided into 4 groups as follows: normal diet fed rats with vehicle (NDV), high-fat diet fed rats with vehicle (HFV), normal diet fed rats with D-gal (NDDg) and high-fat diet fed rats with D-gal (HFDg) rats. For HBOT-treated rats, they were divided into 3 groups as follows: HFV, NDDg and HFDg rats. For HBOT-treated rats, they were put in the hyperbaric chamber and 100% oxygen (O_2_) with 250 L/min flow rate, 2ATA were given to the rats for 60 minutes with pre-10 minutes for compression phase and decompression phase for 10 minutes, total duration: 80 minutes. Regarding sham groups, rats were given oxygenation at ambient pressure with 80 L/min flow rate for 80 minutes. Both HBOT and sham were given to the rats once daily for 14 days [19, 20].

**Figure 10:**
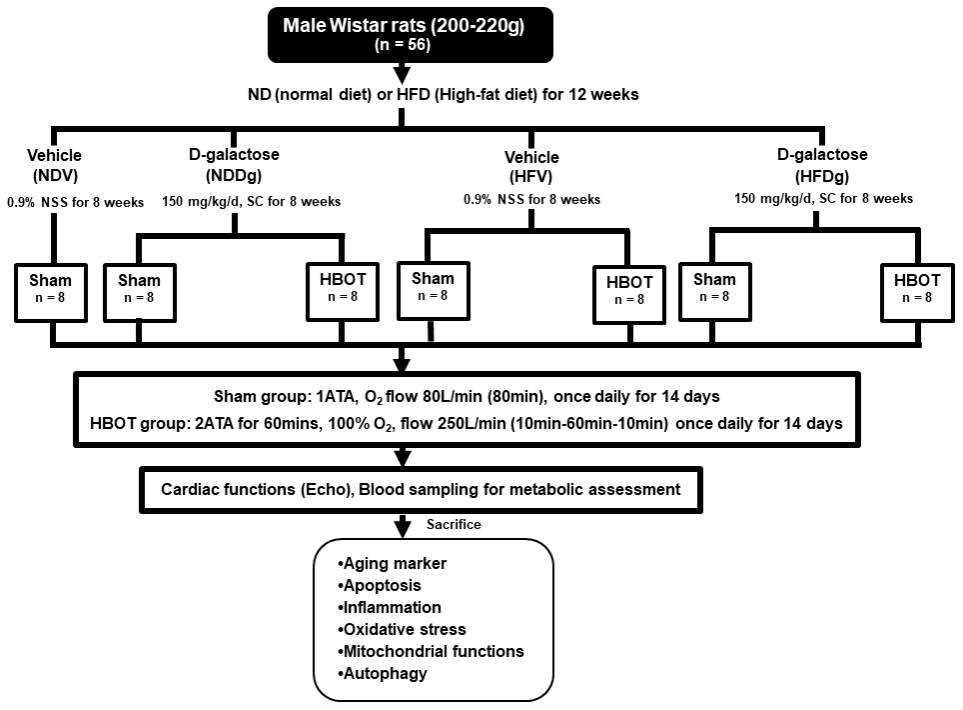
**Study design.** Fifty-six Wistar rats were fed normal diet or high-fat diet for 12 weeks. For subsequent 8 weeks, they were subcutaneously injected either vehicle (0.9% normal saline) or D-gal (150mg/kg/day). Rats were randomly subdivided into 7 groups at week 21: sham-treated (normal diet fed rats with vehicle (NDV), high-fat diet fed rats with vehicle (HFV), normal diet fed rats with D-gal (NDDg), high-fat diet fed rats with D-gal (HFDg)) and HBOT-treated (HFV, NDDg, HFDg). Sham rats received ambient pressure of oxygen while HBOT-treated ones received 100% oxygen given once daily for 60 minutes at 2 ATA. ND, normal diet; HFD, high-fat diet; SC, subcutaneous; NSS, normal saline; NDV, normal diet fed rats with vehicle; NDDg, normal diet fed rats with D-gal; HFV, high-fat diet fed rats with vehicle; HFDg, high-fat diet fed rats with D-gal, ATA, atmosphere absolute; HBOT, hyperbaric oxygen therapy.

Throughout the experimental period, body weight and food intake of all rats were recorded. About 24 hours after sham and HBOT treatment, blood pressure measurement, echocardiography and heart rate variability for determining autonomic activity were performed. Blood was collected from the rats’ tail veins for metabolic assessment. Rats were anesthetized using Zoletil (50 mg/kg) and Xylazine (0.15 mg/kg) via intramuscular injection at the end of the study protocol, and a pressure-volume (P-V) loop recording system was used to determine left ventricular (LV) function. Rats were then sacrificed and mitochondrial function and biochemical studies were determined after the removal of the heart. The experimental design is illustrated in Figure 10. Heart tissues from 8 rats/group were utilized for determining the cardiac and mitochondrial functions. As regard to senescence associated β galactosidase and TUNEL staining, heart tissues from 3 rats/group were determined. For the rest of the other parameters, heart tissues from 5 rats/group were utilized.

### Metabolic parameters assessment

Plasma glucose and triglyceride (TG) levels were detected by colorimetric assay using a commercially available kit (Biotech, Bangkok, Thailand). Plasma insulin level was evaluated by a sandwich ELISA kit (Millipore, MI, USA). Insulin resistant degree was evaluated by homeostasis model assessment (HOMA); which is determined from the formula using fasting plasma glucose and insulin levels. Fasting plasma high-density lipoprotein (HDL) and total cholesterol (TC) levels were assessed with the commercially available kit purchased from ERBA diagnostic, Mannheim, Germany [14].

### Tail-cuff blood pressure determination and assessment of echocardiography

Systolic blood pressure (SBP), diastolic blood pressure (DBP) and mean arterial pressure (MAP) were obtained from the non-invasive CODA2 channel system according to our recent study [14].

Echocardiography, non-invasive method, was used to assess LV function. Rats received light anesthesia with the use of isoflurane 2% with 2 L/min of O_2_. An echocardiography probe was placed at the parasternal short axis of the chest, which was connected to a machine. At the papillary muscles, M mode echocardiography was determined. Percent ejection fraction (%EF) and % fractional shortening (%FS) were evaluated [14].

### Assessment of heart rate variability (HRV)

HRV was assessed via restraining the limbs of the rats under inhalational anesthesia (2.5% isoflurane) in a prone position. A needle electrode was subcutaneously placed into the limbs. When the rats gained full consciousness, ECG recording was performed. 20 minutes of ECG signals were recorded by using signal transducer, and functioned via a Chart 5.0 program. For HRV data analysis, selection of 300 consecutive RR intervals obtained from tachogram was performed. LF and HF from HRV were determined and an increase in LF/HF ratio demonstrates the imbalance of cardiac sympathovagal activity [14].

### Assessment of pressure-volume (P-V) loop study

P-V loop study was performed and analyzed following the steps as reported in our recent study [14]. The investigated parameters gained from the P-V loop study involved end-systolic pressure (ESP), end-diastolic pressure (EDP), maximum and minimum dP/dt (dP/dtmax and dP/dtmin), and heart rate (HR).

### Evaluation of senescence-associated β-galactosidase (SA-β-gal) staining

SA-β-gal staining was determined following the manufacturer’s instructions (Cell Signaling Technology) and the steps shown in our previous study [14]. The senescent cells were detected as blue precipitated cells in the cytoplasm with magnification of x200 [8]. SA-β-gal positive cells numbers were determined in three fields using Image J software as previously described in a recent study [14].

### Cardiac mitochondrial function assessment

Cardiac mitochondrial functions were evaluated by assessing mitochondrial ROS production, mitochondrial membrane potential changes, and mitochondrial swelling following the steps outlined in our recent report [14]. By using transmission electron microscope (TEM), mitochondrial morphology from cardiac tissue was evaluated [14].

### Assessment of cardiac mitochondrial dynamics

Regarding the dynamic processes of mitochondria, western blot analysis was performed in order to detect the expressions of the mitochondrial fusion proteins; mitofusin 1/2 (MFN1/2), and fission protein; mitochondrial dynamin-related protein 1 (Drp1) from the isolated crude mitochondrial fraction and the cytosolic phosphorylated Drp1expression according to the steps as reported in our recent study [14].

### Assessment of cardiac oxidative stress and inflammation

Oxidative stress in cardiac tissue was assessed by malondialdehyde (MDA) level with the use of high-performance liquid chromatography (HPLC) system and inflammation in heart tissue was evaluated with the use of an ELISA kit as shown in a previous study [14] according to the protocol from manufacturer (Thermo Fisher Scientific).

### TUNEL assay for cardiomyocyte apoptosis quantification

Cardiomyocyte apoptosis was evaluated by Terminal deoxynucleotidyl transferase nick-end labeling (TUNEL) positive nuclei colocalized with DAPI staining with the use of *in situ* cell death detection kit. Percent of the number of TUNEL-positive apoptotic cells divided by the total number of DAPI stained cells (nucleated cells) was determined as the apoptosis index as described in previous studies [14, 45, 46].

### Cardiac expression of apoptotic and autophagy proteins

Western blot analysis was carried out for measuring the expression of proteins; Bax, Bcl-2, caspase 3, and Cleaved-caspase 3, p62, Beclin 1, LC3II using anti- p62, Beclin 1, LC3II, caspase 3, Cleaved-caspase 3 and anti-GAPDH. Bound antibody was detected by the use of horseradish peroxidase conjugated with anti-rabbit or anti-mouse IgG. For visualization of the peroxidase reaction products, ECL detection reagent was used [14].

### Statistics

Data have been expressed as mean ± standard error of mean (SEM). Comparisons of variables among the groups were evaluated with the use of one-way-ANOVA with LSD post-hoc test and statistical significance was considered at P value < 0.05.
